# Skeletal muscle ATP kinetics during exercise in patients with systolic heart failure

**DOI:** 10.1186/1532-429X-15-S1-O29

**Published:** 2013-01-30

**Authors:** Gurusher S Panjrath, Michael Schär, AbdEl-Monem El-Sharkawy, Refaat Gabr, Steven P Schulman, Gary Gerstenblith, Paul A Bottomley, Robert G Weiss

**Affiliations:** 1 Cardiology, The Johns Hopkins University School of Medicine, Baltimore, MD, USA; 2 Radiology/MR research, The Johns Hopkins University School of Medicine, Baltimore, MD, USA

## Background

Creatine kinase (CK) reaction is the primary energy reserve reaction in skeletal muscle (SM), providing additional ATP during exercise. Although exercise intolerance is a major limitation in systolic heart failure (SHF), the rate of ATP delivery by CK (CK flux) has not been studied during exercise in healthy subjects (HS) or SHF patients. We hypothesized that SM high-energy phosphates and CK flux are reduced at rest and during low intensity plantar flexion exercise (PFE) in mild-moderate SHF patients.

## Methods

14 non-ischemic SHF patients (EF<40) and 11 HS underwent 31P magnetic resonance spectroscopy with saturation transfer (MRST) measures of gastrocnemius creatine phosphate-to-ATP (PCr/ATP) and CK pseudo-first-order rate constant, k, at rest and during PFE in a Philips 3T MRI/MRS system. Single-leg PFE involved lifting 0.9 kg/sec on a custom built non-magnetic device for 8.5 minutes. [ATP](umol/g) was taken as 5.2umol/g for both groups and [PCr](umol/g) was calculated as (PCr/ATP)*(5.2umol/g) and CK flux (umol/g/s) as ([PCr]*k). Comparisons were made with paired and non-paired t-testing using XLSTAT package.

## Results

In HS, baseline PCr/ATP, k and CK flux were 4.4±1.7, 0.27±0.04s-1 and 6.4±3.2umol/g/s, (mean±SD) respectively. All subjects completed the 8.5 min PFE. SM PCr declined during early exercise and stabilized at new level during MRST. During PFE in HS, mean [PCr] decreased ~20%, k increased ~31%, and CK flux was unchanged (table 2). Baseline energetic indices were not lower but trended non-significantly higher in SHF. During PFE although CK flux decreased significantly as compared to baseline (p<0.04), it was not below that in HS.

**Table 1:** Baseline Characteristics and Exercise Capacity

	Healthy (n=11)	SHF (n=14)
Age (yrs)	41.5 ± 11.1	50.8 ± 9.5*
Male %	82	71
BMI	25 ± 2.7	31 ± 6.0 ¶
NYHA Class	-----	1.9 ± 0.7
LVEF (%)	-----	24 ± 9
Peak VO2 (ml/kg/min)	27.6 ± 2.3	18.7 ± 6.0 ¶
6MWD (ft)	1683 ± 258	1156 ± 246 ¶

BMI- body mass index; 6MWD-six minute walk distance, mean±SD * p <0.05; ¶ p< 0.01 compared to healthy

**Table 2:** Phosphate Metabolites and Creatine Kinase Flux at Rest and During Plantar Flexion Exercise

	Healthy (n=11)	SHF (n=14)
PCr/ATP (rest)	4.42 ± 1.67	5.70± 2.0
PCr/ATP (ex)	3.76 ± 1.73¶	4.46± 2.2 ¶
k (s-1)(rest)	0.27 ± 0.04	0.29 ± 0.05
k (s-1)(ex)	0.35 ± 0.11 *	0.35 ± 0.06 *
PCr (µmol/g) (rest)	22.9 ± 8.7	29.4 ± 10.9
PCr (µmol/g)(ex)	18.8 ± 9.7 §	22.2 ± 9.8 §
CK flux (µmol.g-1.s-1)(rest)	6.37 ± 3.15	8.53 ± 2.96
CK flux (µmol.g-1.s-1) (ex)	6.68 ± 4.44	7.57 ± 2.62*

PCr/ATP-phosphocreatine/Adenosine triphosphate ratio; k- CK reaction pseudo first order rate constant; mean±SD, * <0.05 compared to rest; ¶ <0.01 compared to rest, § <0.001 compared to rest

## Conclusions

The novel findings indicate that (1) in HS despite a decline in [PCr] and PCr/ATP during exercise, CK flux is maintained with increase in k, (2) at baseline, SM PCr/ATP and CK flux are not lower in HF than in HS and (3) ATP flux through CK during exercise is unchanged from baseline in HS but declines significantly in SHF, (4) despite the decline, CK flux in SHF is not lower than that in HS. Although SM CK flux falls during exercise in SHF it appears that exercise-induced changes in SM CK ATP kinetics, at least with low-intensity exercise, may not be limiting in mild-moderate SHF.

## Funding

NIH HL61912 and Clarence Doodeman Endowment.

